# Conformational dynamics and energetics of viral RNA recognition by lab-evolved proteins†

**DOI:** 10.1039/d1cp03822b

**Published:** 2021-10-29

**Authors:** Amit Kumar, Harish Vashisth

**Affiliations:** Department of Chemical Engineering, University of New Hampshire Durham 03824 New Hampshire USA harish.vashisth@unh.edu

## Abstract

The conserved and structured elements in viral RNA genomes interact with proteins to regulate various events in the viral life cycle and have become key targets for developing novel therapeutic approaches. We probe physical interactions between lab-evolved proteins and a viral RNA element from the HIV-1 genome. Specifically, we study the role of an arginine-rich loop in recognition of designed proteins by the viral RNA element. We report free energy calculations to quantitatively estimate the protein/RNA binding energetics, focusing on the mutations of arginine residues involved in recognition of the major groove of RNA by proteins.

## Abstract 2

The conserved and structured elements in viral RNA genomes interact with proteins to regulate various events in the viral life cycle and have become key targets for developing novel therapeutic approaches.

## Graphical abstract

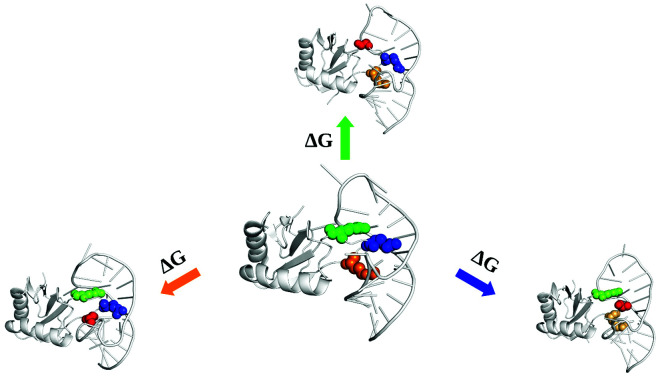

## 1 Introduction

RNA viruses, including influenza, Ebola, HIV, and SARS-CoV-2, are among the most efficient and compact carriers of biological information in nature,^1^ and are implicated in many ongoing and emerging threats to human health.^2–5^ The highly compact and short genomes of these viruses provide a limited number of protein targets for anti-viral therapeutics because of the lack of well-defined binding pockets.^6^ However, the conserved and structured RNA motifs in viral genomes direct various events, often through their interactions with RNA binding proteins, during infection including genome packaging, replication, regulation of protein expression, and evading degradation by the host cell.^7–11^ Therefore, these viral RNA motifs and RNA–protein interfaces are promising targets for anti-viral drug discovery.

However, RNA–protein interactions often span a large surface area, which makes it difficult to target them with small molecules.^12–17^ By virtue of their size, peptides can often bind to RNA with excellent potency and may modulate the biological function.^18,19^ Many RNA binding proteins exploit arginine-rich β-hairpin structures to recognize RNA molecules.^20,21^ Therefore, β-hairpin peptides are of particular interest for targeting viral RNA molecules. Although small β-hairpin peptides that potently and selectively recognize viral RNA molecules are rare and *de novo* design of these molecules is an unresolved challenge,^22,23^ advances in protein engineering have facilitated the design and screening of lab-evolved RNA binding peptides with predefined functions and specificity.^24^ Moreover, a recent study by Shortridge *et al.*^25^ also demonstrates how macrocyclic peptides could be synthesized with ultra-high affinity for the RNA. However, the mechanisms by which lab-evolved peptides recognize disease-relevant RNAs are poorly understood.

**Fig. 1:**
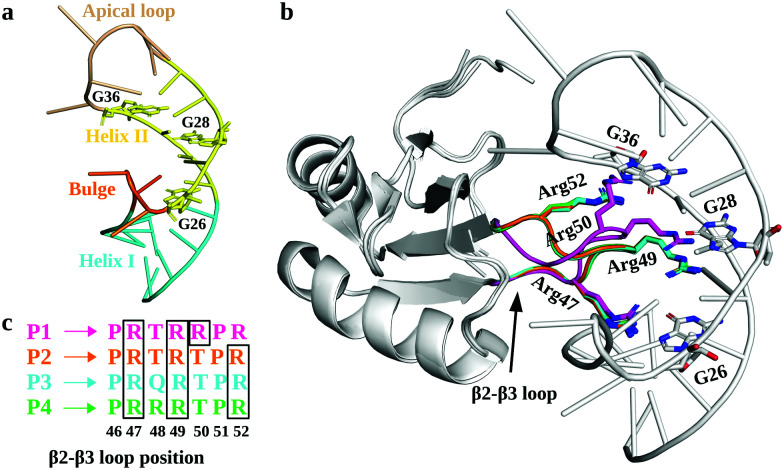
Structural details of TAR and its complexes with TBPs. (a) The tertiary structure of HIV-1 TAR RNA (PDB: 6CMN) is shown with different structural motifs (Helix I, Helix II, Bulge, and Apical loop) uniquely colored and labeled. The key nucleotides involved in TAR interactions with TBPs are shown in sticks. (b) A cartoon representation of superimposed TBPs bound to TAR RNA. The β2–β3 loop residues involved in the recognition of TAR and subjected to mutations are labeled and shown with sticks. (c) The lab-evolved β2–β3 loop sequences from different TBPs and the arginine residues involved in the TAR major groove binding are shown in the boxes.

In this regard, RNA motifs from the HIV genome^26–28^ serve as good model systems^29–31^ to understand the viral RNA recognition mechanism by lab-evolved proteins. The HIV-1 *trans*-activation response element RNA (TAR; Fig. 1a), a 59-nucleotide long conserved RNA element located at the 5′ end of the HIV-1 transcripts, interacts with the viral transactivator (Tat) protein and the host cofactor cyclin T1 to regulate the viral RNA production.^32–35^ Therefore, inhibiting TAR may disrupt the viral replication process and prove useful in design of novel therapies.^36–38^

**Fig. 2:**
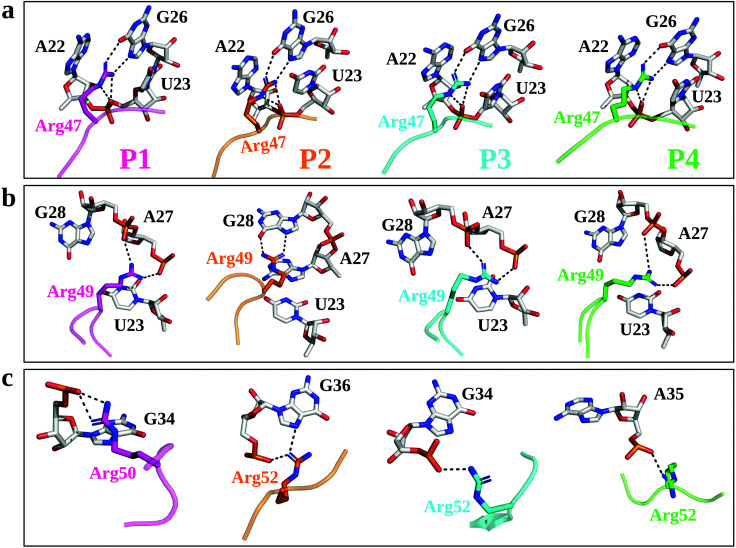
Structural insights from MD simulations of the TBP–TAR complexes. Key TBP residues and TAR nucleotides are represented by sticks. The interaction pattern of key Arg residues (Arg47, panel (a); Arg49, panel (b); and Arg50 or Arg52, panel (c)) of TBPs with TAR major groove are shown. The averaged structure of each TBP is shown (pink, P1; orange, P2; cyan, P3; green, P4). The interactions in the major groove of TAR are shown with the black dotted lines.

The crystal structures of TAR in complexes with lab-evolved TAR binding protein (TBP) variants TBP6.3 (P1), TBP6.6 (P2), TBP6.7 (P3), and TBP6.9 (P4) have been reported (Fig. 1b).^39,40^ These proteins were evolved with 3 or 4 Arg residues (P1 and P4 have 4 Arg residues; P2 and P3 have 3 Arg residues) at distinct positions within the β2–β3 loops of TBPs (Fig. 1c). Specifically, these structures revealed that TBPs acquire TAR binding specificity through Arg47, Arg49, and Arg52-mediated readout of the Hoogsteen edge of conserved GUA26, GUA28, and GUA36 nucleobases, except in P1, where Arg50 (instead of Arg52 in P2, P3, and P4) recognizes GUA36.^39,40^ Other amino acid residues in the β2–β3 loop promote either phosphate backbone interactions or intramolecular hydrogen bonding that stabilize the backbone in the β2–β3 loop. Further, biochemical studies suggest that variations in Arg composition and placement in the β2–β3 loop alter the affinity of TBPs for TAR. The dissociation constant (*K*_d_) values for TBPs range between 3.0 nM and 45.2 nM.^39–42^

Although the experimental structures have enriched our understanding of viral RNA recognition by lab-evolved proteins (TBPs), to what extent the affinity of TBPs for TAR is affected in response to the mutations in the β2–β3 loop (*i.e.*, relative binding affinity ΔΔ*G*), and how is ΔΔ*G* linked to the structural details of the wild-type (WT) and mutant TBPs remains unknown. Here, computer simulations are useful tools to link the structures and energetics, since the structure-based free energy calculations of mutations can be evaluated with sufficiently high accuracy.^43–45^

Hence, using the crystal structures^39,40^ as our initial models, we have studied the dynamics of the WT TBP–TAR complexes by all-atom explicit-solvent conventional molecular dynamics (MD) simulations (Fig. S1a and Table S1, ESI†) and have reported free energy calculations for deciphering the energetics of TBP mutations (R47A, R49A, and R52A). Specifically, we computed the energetics of mutations *via* the free energy perturbation (FEP) method^46,47^ and quantified the relative changes in the binding affinity of TBPs with TAR using an appropriate thermodynamic cycle (Fig. S1b, ESI†). Aiming to understand the mechanism of TAR recognition by lab-evolved TBPs, we performed cumulative 16.4 µs of MD simulations.

## 2 Materials and methods

### 2.1 Simulation setup

We retrieved the structures of TBP variants TBP6.3 (P1), TBP6.6 (P2), TBP6.7 (P3), and TBP6.9 (P4) bound TAR complexes from the Protein Data Bank (PDB) entries 6XH3, 6XH2, 6CMN, and 6XH0, respectively.^39, 40^ We then changed residues Y31 to H31 and Q36 to R36 because these mutations were introduced to facilitate crystallization. We retained the atomic coordinates of water molecules and ions from the crystal structures and solvated the structures of all complexes with explicit TIP3P^48^ water molecules in a periodic simulation domain (80 Å × 80 Å × 80 Å). We then neutralized each system by placing Mg^2+^ and Cl^−^ ions into the minima of the electrostatic potential computed using the meadionize plugin in VMD.^49^ Finally, we also added K^+^ and Cl^−^ to the bulk water to maintain the 150 mM KCl ionic concentration. The final system sizes are given in Table S1 and the overall simulation setup is shown in Fig. S1a (ESI†).

Before conducting all-atom molecular dynamics (MD) simulations, we minimized each system for 2000 steps by using the conjugate gradient minimization algorithm. During the initial phase (30 ns) of MD equilibration, we restrained (*k* = 10 kcal mol^−1^ Å^2^) the C_α_ atoms for TBPs and the P-atoms in RNA. After this phase, we removed the restraints and performed production MD simulations using the CHARMM36^50^ force field with a 2 fs time-step in the *NPT* ensemble. We maintained the temperature at 300 K using the Langevin thermostat with a coupling coefficient of 5 ps^−1^ and the pressure at 1 bar using the Nose–Hoover barostat. We used periodic boundary conditions and treated long-range electrostatics using the Particle Mesh Ewald method.^51^ We truncated van der Waals interactions at 16 Å with smooth switching taking effect at 15 Å.

For each simulation model (P1–P4/TAR complex), we generated a 2 µs long MD trajectory after the initial equilibration phase. We used VMD for generating input files and NAMDv2.13^52^ for conducting simulations. We carried out the analyses of MD trajectories with VMD^49^ and Pymol.^53^ We performed free energy calculations using a new GPU implementation of the free energy perturbation (FEP) method in NAMDv3.0.^54^

### 2.2 Protocol for binding free energy calculation

We calculated the relative binding free energies (ΔΔ*G*_bind_) for TBP mutations in the TBP-TAR complexes by alchemically transforming the wild-type amino acid into a mutated amino acid using a thermodynamic cycle (Fig. S1b, ESI†). The vertical arms of the cycle correspond to binding of each TBP, and the horizontal arms correspond to the alchemical transformation in a given TBP which cannot be experimentally realized. We computed the free-energy changes along the horizontal arms of the cycle (Δ*G*^comp^ and Δ*G*^free^) and calculated the relative binding free energy as ΔΔ*G*_bind_ = ΔG^comp^ − ΔG^free^ = Δ*G*_bind_ (wild-type) − Δ*G*_bind_ (mutant).

We used a hybrid energy function (*U*) to represent a mixture of two endpoint states of a particular horizontal arm of the thermodynamic cycle (Fig. S1b, ESI†). The coupling parameter *λ* connects the initial (I) and final (F) states by a series of equispaced intermediate states. The coupling parameter values *λ* = 0 and 1, correspond to physical end states, whereas an intermediate value corresponds to a mixed unphysical state. Using the previously described^55^ FEP method, we obtained the total free energy change Δ*G* along the variable horizontal paths by summing over the intermediate states as the following: 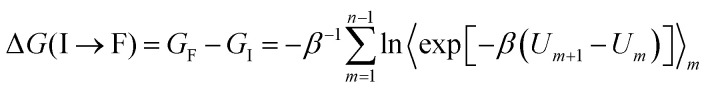, where *U*_*m*_ = (1 − *λ*_*m*_) *U*_I_ + *λ*_*m*_*U*_F_ with the coupling parameter *λ*_m_ varying from 0 to 1, and the total number of intermediate points *m* = 1,…,(*n* − 1). Here, *β* is 1/*k*_B_*T*, with *k*_B_ as Boltzmann*s constant and *T* is the temperature.

**Fig. 3:**
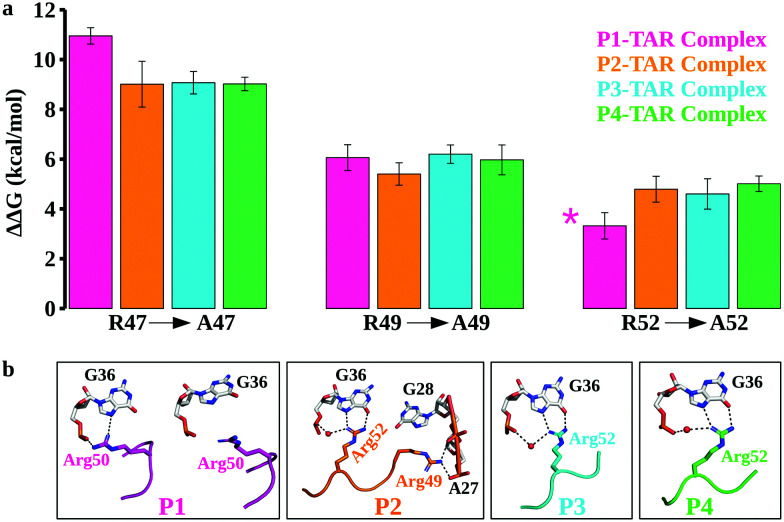
Energetics of TBP binding to TAR and structural changes upon mutations. (a) The relative binding free energy (ΔΔ*G*) of TBP mutations (R47A, R49A, and R52A). In the case of P1 TBP variant, we did calculations for R50A (shown by *). The error bars are represented by the standard error of the mean and binding free energies are in kcal mol^−1^. (b) Structural insights from the TBP–TAR complexes upon R47A and R49A mutations. Key interactions are highlighted by the black dotted lines. Water molecules are indicated by a red sphere.

**Fig. 4:**
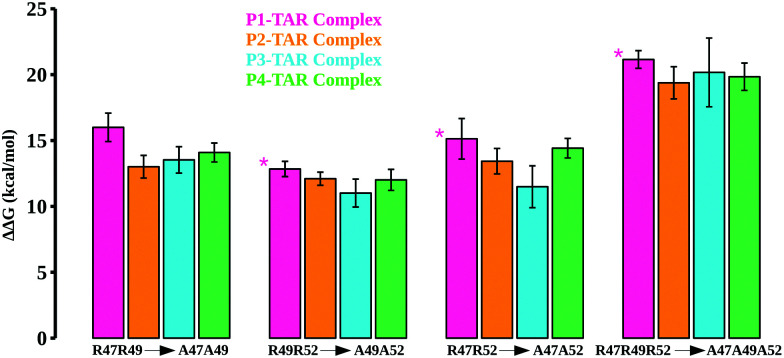
Calculated TAR binding energetics for the double/triple Arg mutations in TBPs. The calculated relative binding free energies for mutant pairs R47A–R49A, R49A–R52A, R47A–R52A, and R47A–R49A–R52A are shown in different colors. For the P1/TAR complex, we carried out calculations for R50A (shown by *).

In our free energy calculation protocol, “alchDecouple” is set to “OFF” to scale the nonbonded interaction along the alchemical coordinate. This protocol allowed us to scale the nonbonded interactions of the mutated residue with their environment and within the mutated residue, which contribute to the cumulative free energy. We averaged the total free energy change (Δ*G*^free^, Δ*G*^comp^) over forward and backward simulations with 25 equally spaced *λ*_*m*_ values and repeated in triplicate, yielding a minimum of 150 ns of simulation data per transformation (Tables S2–S5 and S7–S10, ESI†). We initiated each replica using different initial velocities and have reported the difference of the averaged Δ*G*^comp^ and Δ*G*^free^ as ΔΔ*G* (Fig. 3a and 4).

We used the bidirectional approach to estimate the free energy differences by incorporating samples from both forward and backward transformations. We estimated the free energy difference and the associated statistical error using the Bennet acceptance-ratio (BAR) estimator implemented in the ParseFEP toolkit^56^ of VMD. We compared the graphical representation of the underlying probability distributions characterizing the forward and backward transformations to ensure the convergence. We obtained a good convergence and a reasonable statistical uncertainty (<1 kcal mol^−1^) of the computed energetics (ΔΔ*G*) for single Arg-to-Ala mutation. However, we observed hysteresis upon simultaneous mutations of two or three Arg residues in the TBP-TAR complexes during forward (R → A) and reverse (A → R) alchemical transformations. We have observed a large conformational change in the β2–β3 loop of TBPs upon Arg-to-Ala mutations in combinations of two or three during forward transformation (R → A). Therefore, the β2–β3 loop adopts a different conformation than the one in the crystal structure upon reverse transformation (A → R, see Fig. S2, ESI†), leading to hysteresis in calculated free energies. Hence, we averaged the free energy values for double or triple Arg mutations over three replicas (25 ns each) only from the forward simulations (Fig. 4).

## 3 Results

### 3.1 Dynamics of TBP–TAR complexes

We first used µs-scale MD simulations to investigate the conformational changes in the TBP–TAR complexes. Overall, the MD-equilibrated structures of the TBP–TAR complexes are stable with the average root mean squared deviation (RMSD) values of the non-hydrogen atoms as (Fig. S3a, ESI†): 5.66 ± 1.1 Å (P1/TAR), 7.29 ± 1.7 Å (P2/TAR), 4.9 ± 0.77 Å (P3/TAR), and 5.16 ± 1.59 Å (P4/TAR). Our calculated average RMSD values (Fig. S3b, ESI†) suggest that the TAR RNA contributes the most to the overall RMSD of the complex and the average RMSD values of the non-hydrogen atoms of TAR from four complexes are: 8.15 ± 1.66 Å (P1/TAR), 10.56 ± 2.68 Å (P2/TAR), 6.8 ± 1.2 Å (P3/TAR), and 7.28 ± 1.34 Å (P4/TAR). To gain insights into the dynamics of individual residues in the TBP–TAR complexes, we have calculated the average RMSD per residue (of non-hydrogen atoms) with respect to the X-ray structure for both TAR and TBPs (Fig. S4, ESI†). These data reveal larger average RMSD values for the TAR RNA. The average RMSD is higher for the apical loop residues (URA31, GUA32, and GUA33) and bulge residues (CYT24 and URA25) of TAR (Fig. S4a, ESI†). However, MD simulations further suggest that the URA23–ADE27–URA38 base triplex and the CYT30–GUA34 canonical base pair, which are hallmark features of the ligand-bound TAR conformation in solution, remain intact during simulations, except in the P4/TAR complex where the CYT30–GUA34 base pair is disrupted (Fig. S5, ESI†).

MD simulations suggest that Arg47 makes most stable and extensive interactions with TAR in all four TBP–TAR complexes (Fig. 2a and Fig. S6, S7, ESI†). The side-chain of Arg47 is stacked between URA23 and ADE22 nucleobases and forms a salt-bridge to URA23 (non-bridging phosphate oxygen), which locks the conformation of Arg47 so that its side-chain makes hydrogen bonds with the Hoogsteen edge of GUA26 (Fig. 2a). The side-chain of Arg49 stacks on the URA23 nucleobase and forms salt-bridges with the non-bridging phosphate oxygen of ADE27 and GUA28 except in the P2/TAR complex where it stacks on ADE27 and forms H-bonds with the Hoogsteen edge of GUA28 (Fig. 2b). Further, we observed that the side-chain of the third arginine residue (Arg50 for P1 and Arg52 for P2, P3, and P4) salt bridges to GUA34 in P1 and P3, GUA36 in P2, and ADE35 in P4 (Fig. 2c). The side-chain of Arg52 in the P2/TAR complex also interacts with the Hoogsteen edge of GUA36. Our results suggest that the interactions of the third arginine (Arg50 for P1 and Arg52 for P2, P3, and P4) with TAR are transient (see Fig. S7, ESI†). The fractional occupancy calculations also demonstrate that the Arg49 and Arg52 residues in some cases make hydrogen bonds with the Hoogsteen edge of GUA28 and GUA36, respectively, but the average occupancies of their salt-bridge interactions are dominant, as shown in Fig. S7 (ESI†). The pattern of interactions observed for all TBP-TAR complexes in this study agrees well with a previous study of the P3/TAR complex carried out with the AMBER interatomic potential, confirming that conformational shifts on the sites Arg49 and Arg52 sites are purely mechanistic observations.^39^

### 3.2 Energetics of arginine mutations (R47A, R49A, and R52A) and its effect on the TBP–TAR Complexes

While conventional MD simulations suggest that Arg47, Arg49, and Arg52 (Arg50 in case of P1) in the β2–β3 loop of TBPs participate in recognition of the major groove of TAR, they do not quantify the binding contributions of these residues. To compute the energetics and the effect of arginine mutations (R47A, R49A, and R52A), we performed extensive FEP calculations of the TBP–TAR complexes. We computed the change in the TBP–TAR binding affinity upon an Arg to Ala mutation in two ways: (i) by mutating one Arg to Ala at a time in each complex and (ii) by mutating Arg to Ala in combination of two or three Arg residues together. We then quantified the relative binding affinity changes upon Arg to Ala mutations using a thermodynamic cycle (Fig. S1b, ESI†).

The calculated relative changes in binding affinities upon a single Arg to Ala mutation (Tables S2–S5, ESI†) for different TBP–TAR complexes are summarized in Fig. 3a. We make the following observations: (i) the R47A mutation in P1 imposes the highest energetic penalty of ∼11 kcal mol^−1^ while the same mutation in P2, P3, and P4 penalizes by ∼9 kcal mol^−1^ for TAR recognition relative to WT TBPs; (ii) the R49A mutation in all four TBPs reduces the TAR binding affinity by ∼6 kcal mol^−1^ relative to WT TBPs, and (iii) the R52A mutation in P2, P3, and P4 variants reduces the TAR binding affinity by ∼5 kcal mol^−1^, while the corresponding R50A mutation in P1 imposes the least energetic penalty of ∼3.3 kcal mol^−1^ relative to the WT TBPs. An earlier biochemical study of Arg-to-Ala mutation in P3 suggested a larger energetic penalty for R47A and the least energetic penalty for R52A.^39^ Consistent with this biochemical study (Table S6, ESI†), we observed a similar pattern of TBP–TAR binding discrimination in our computed ΔΔ*G* values upon Arg-to-Ala mutations in the TBP–TAR complexes (see Fig. 3a).

We observed that the R47A mutation results in disruption of interactions between the side-chain of Arg47 and TAR. The Arg49 interactions remain intact except in the P2/TAR complex where the side-chain of Arg49 orients toward the backbone phosphate of ADE27 and GUA28, and establishes salt-bridging interactions (Fig. 3b and Fig. S8, ESI†). It is interesting to note that upon R47A mutations, the side-chain of Arg52 (Arg50 in P1) interacts with the Hoogsteen edge of GUA36 and also forms a water-mediated or direct interaction with its phosphate oxygen (Fig. 3b). We have also observed similar interactions of Arg52 with TAR upon R49A mutation except in the P1/TAR complex wherein the side-chain of Arg50 is reoriented away from the GUA36 (Fig. 3b and Fig. S9, ESI†). Furthermore, upon R52A (R50A in P1) mutation, we have observed that the side-chain of Arg47 and Arg49 interacts with TAR in a similar manner to as observed for R49A and R47A mutations, respectively (Fig. S10, ESI†).

We also mutated key Arg residues in combinations of two (mutant pairs R47A–R49A, R49A–R52A, and R47A–R52A) and three mutations (mutant R47A–R49A–R52A) together to see the differences in binding affinities and conformational changes in the TBP–TAR complexes beyond those observed for single Arg to Ala mutations. The computed energetics show an incremental loss in binding affinities of TBPs in the TBP–TAR complexes (Fig. 4 and Tables S7–S10, ESI†). Relative to the WT TBPs, we observed a significant loss in binding affinity (ranging from ∼19 kcal mol^−1^ to ∼21 kcal mol^−1^) upon mutations in all three key Arg residues (Tables S7–S10, ESI†).

The mutations R47A–R49A (Fig. S11, ESI†), R49A–R52A (Fig. S12, ESI†), R47A–R52A (Fig. S13, ESI†), and R47A–R49A–R52A (Fig. S14, ESI†) together result in disruption of interactions between key Arg residues and TAR. We observed that upon R47A–R49A–R52A triple mutations, only Arg52 of P1 and Arg48 of P4 remain in the proximity of TAR nucleotides URA23 and GUA36, respectively. We note that the mutants where Arg47 substitution is not involved (*e.g.*, single mutants R49A and R52A, and double mutant R49A–R52A), the interactions of Arg47 with URA23 and GUA26 remain intact, thus implicating Arg47 in the stability of the TBP–TAR complexes.

## 4 Discussion

Biochemical and structural studies^39–41^ suggest that two TBPs, P2 and P3, are identical in their evolved β2–β3 loop except that the residue Thr48 is replaced by Gln48 in P3 (Fig. 1c). Also, the crystal structures suggest that the recognition of TAR by these TBPs is similar with a subtle variation in the interactions of Thr48 and Gln48 leading to one less H-bond with TAR in P2. However, biochemical studies suggest that P2 (*K*_d_ = 4.2 nM) binds TAR with a slightly better affinity than P3 (*K*_d_ = 5.3 nM) due to the Thr48–Thr50 intrapeptide interactions that stabilize the β2–β3 loop in P2. Our free energy calculations suggest that the Arg-to-Ala mutations reduce the TAR binding affinity of P2 and P3 with nearly the same magnitude, except that the ΔΔ*G* values for the R49A mutation are ∼5.4 kcal mol^−1^ (P2/TAR) and ∼6.2 kcal mol^−1^ (P3/TAR) (see Fig. 3a). Our results show that this energetic difference in the R49A mutation is due to salt-bridging interactions between the side-chain of Arg49 (P3) and TAR nucleotides compared to hydrogen bonding of Arg49 with the Hoogsteen edge of GUA28 in P2. Further, our conventional MD simulations revealed that the side-chain of Thr50 in P2 interacts with the Hoogsteen edge of GUA34 and the atom NH1 in the guanidinium group of Arg52 (Fig. S6, ESI†). The backbone of Thr50 also forms hydrogen bonds with the side chains of Thr48 and Arg52 and stabilizes the β2–β3 loop conformation (Fig. S6, ESI†). Therefore, our results suggest that threonines not only form intrapeptide interactions to stabilize the β2–β3 loop, but they also interact with TAR and contribute to the overall TAR binding affinity of P2, giving it a marginally higher preference for binding to TAR than P3.

Two TBPs, P1 and P4, were evolved with 4 Arg residues in their β2–β3 loop and only 3 Arg residues are involved in the recognition of conserved TAR guanines.^40,41^ However, biochemical studies showed a difference in P1 (*K*_d_ = 45.2 nM) and P4 (*K*_d_ = 3.0 nM) binding affinities for TAR. Our free energy calculations suggest that the R49A mutation in P1 and P4 reduces the TAR binding affinity with about the same magnitude, ΔΔ*G* = ∼6 kcal mol^−1^ (see Fig. 3a). We observed a large difference in the energetics of P1/TAR and P4/TAR complexes upon R47A and R52A mutations. The Arg47 residue has the same interaction pattern in both P1/TAR and P4/TAR complexes (Fig. 2a), yet the R47A mutation showed a higher energetic penalty for P1 (ΔΔG values for P1 and P4 are ∼11 kcal mol^−1^ and ∼9 kcal mol^−1^, respectively). Our results suggest that the energetic difference upon R47A mutation in P1 and P4 is due to a distinct placement of 4 Arg residues in the β2–β3 loop (Fig. S6, ESI†).

We observed that the residues Arg47 and Arg49 along with the fourth arginine at position 52 of P1 pull the β2–β3 loop away from the GUA36 nucleotide, leading to more stable Arg47 interactions and transient Arg50 salt-bridging interactions with TAR (Fig. S6 and S7, ESI†), which produces a ΔΔ*G* of ∼3.3 kcal mol^−1^ upon R50A mutation (Fig. 2a). The residues Arg47 and Arg49 of P4 interact with the major groove nucleotides of TAR whereas the remaining two Arg residues (Arg48 and Arg52) form salt bridging interactions with the nucleotides in the Helix II and the apical loop which distributes the positive charge uniformly in the major groove of TAR. This uniform distribution of positively charged residues leads to a stable P4–TAR complex and gives a ΔΔ*G* of ∼5 kcal mol^−1^ upon R52A mutation. Our work suggests that Thr50 of P4 is involved in interactions with TAR and contributes to its overall binding affinity whereas Thr48 of P1 does not form any interaction. Our conventional MD simulations and free energy calculations suggest that the position of Arginine and Threonine is critical to the stability of the β2–β3 loop and its overall binding affinity to TAR.

Further, it is important to note that the nucleotide GUA34 in the P4–TAR complex is oriented away from the major groove of TAR, thereby disrupting the canonical CYT30–GUA34 base-pairing (Fig. S5, ESI†). The previous studies^57,58^ suggested that the CYT30–GUA34 cross-loop pairing is essential for the binding of Cyclin T1, a cofactor of the Tat protein, and for the HIV-1 replication. Our results suggest that P4 not only binds to the TAR major groove, it can also disrupt the base-pairing in the TAR apical loop and can block binding of both Tat protein and its partner protein Cyclin T1. Consistent with our observations of P4–TAR binding, experimental studies have also shown that a small cyclic peptide derived from the β2–β3 loop of P4 inhibits the binding of the Tat protein to major groove of TAR.^40^

## 5 Conclusions

In conclusion, we have provided insights into the dynamics of the TBP–TAR complexes and have reported free energy calculations for quantitatively deciphering the energetics of TBP mutations, thereby linking structural and energetic details. Our results reveal that the Arg47 from the β2–β3 loop recognizes the Hoogsteen edge of the conserved GUA26. In contrast, residues Arg49 and Arg52 (Arg50 in P1) make stable salt-bridging interactions with the phosphate oxygen of TAR. Thus, we suggest a different mechanism of TAR recognition by TBPs than suggested earlier^39,40^ where the Hoogsteen edge of three conserved guanine nucleotides (GUA26, GUA28, and GUA36) are being recognized by three arginine residues in the β2–β3 loop of lab-evolved TBPs. Our simulations reveal that the placement of arginine residues in the β2–β3 is important for uniform distribution of positive charge in the major groove of TAR and the stability of the β2–β3 loop. We also observed that threonine residues form intrapeptide interactions to stabilize the β2–β3 loop and interact with the TAR nucleotides to contribute to the overall binding affinity of TBPs. Therefore, our work provides a clue to the positioning of arginines and threonines in the β2–β3 loop of lab-evolved TBPs for efficient recognition of TAR RNA. Our results are potentially useful for understanding the recognition of other viral RNAs by lab-evolved proteins and in designing a new class of inhibitors targeting TAR.

## Author contributions

A. K. and H. V. designed the research. A. K. performed the research and analyzed data. A. K. and H. V. wrote the article.

## Conflicts of interest

There are no conflicts to declare.

## Supplementary Material

CP-023-D1CP03822B-s001
