# Supplementary material for: Conformational dynamics and energetics of viral RNA recognition by lab-evolved proteins
Source: Phys Chem Chem Phys. 2021 Oct 29;23(43):24773–9. doi: 10.1039/d1cp03822b (PMC8579469; doi:10.1039/d1cp03822b)
Supplement: CP-023-D1CP03822B-s001 [file CP-023-D1CP03822B-s001.pdf]

Electronic Supplementary Material (ESI) for Physical Chemistry Chemical Physics.
This journal is © the Owner Societies 2021

ELECTRONIC SUPPLEMENTARY INFORMATION

Conformational Dynamics and Energetics of Viral RNA Recognition by Lab-evolved Proteins

Amit Kumar and Harish Vashisth*

Department of Chemical Engineering, University of New Hampshire, Durham 03824, New Hampshire, United States

E-mail: harish.vashisth@unh.edu

Table of Contents

Table S1 ………………………………………………………………………………. S3

Table S2 ………………………………………………………………………………. S4

Table S3 ………………………………………………………………………………. S4

Table S4 ………………………………………………………………………………. S5

Table S5 ………………………………………………………………………………. S5

Table S6 ………………………………………………………………………………. S6

Table S7 ………………………………………………………………………………. S7

Table S8 ………………………………………………………………………………. S7

Table S9 ………………………………………………………………………………. S8

Table S10 ………………………………………………………………………………. S8

Fig. S1 ………………………………………………………………………………. S9

Fig. S2 ………………………………………………………………………………. S10

Fig. S3 ………………………………………………………………………………. S11

Fig. S4 ………………………………………………………………………………. S12

Fig. S5 ………………………………………………………………………………. S13

Fig. S6 ………………………………………………………………………………. S14

Fig. S7 ………………………………………………………………………………. S15

Fig. S8 ………………………………………………………………………………. S16

Fig. S9 ………………………………………………………………………………. S17

Fig. S10 ……………………………………………………………………………… S18

Fig. S11 ……………………………………………………………………………… S19

Fig. S12 ……………………………………………………………………………..... S20

Fig. S13 ……………………………………………………………………………… S21

Fig. S14 ……………………………………………………………………………… S22

Table S1. Details of systems for conventional MD simulations.

System	Number of Atoms	Ions		
		Mg^{2+}	Na^{+}	Cl^{-}
P1/TAR	47968	9	42	42
P2/TAR	47959	10	42	43
P3/TAR	47927	10	42	43
P4/TAR	47939	9	42	42

Table S2: Free energy data for single mutations in the TBP variant P1. The computed free energy changes upon R47A, R49A, and R50A mutations of P1 in solution (ΔG^{free}) and in complex (ΔG^{comp}) with TAR. The relative binding free energy ($\Delta\Delta G$) for each mutation is reported in the last column.

**System P1/TAR**	**Runs**	**Simulation Length**	**ΔG^{free}**	**ΔG^{comp}**	**$\Delta\Delta G = (\Delta G^{comp} - \Delta G^{free})$**
R47→A47	Run1	50 ns	264.50 ± 0.11	275.18 ± 0.22	10.95 ± 0.33
	Run2	50 ns	264.24 ± 0.12	274.63 ± 0.25	
	Run3	50 ns	263.98 ± 0.12	275.75 ± 0.26	
		**Average**	**264.24 ± 0.15**	**275.19 ± 0.30**	
R49→A49	Run1	50 ns	264.54 ± 0.13	271.17 ± 0.20	6.06 ± 0.52
	Run2	50 ns	264.13 ± 0.12	270.21 ± 0.22	
	Run3	50 ns	263.90 ± 0.10	269.36 ± 0.20	
		**Average**	**264.19 ± 0.18**	**270.25 ± 0.47**	
R50→A50	Run1	50 ns	265.12 ± 0.12	267.45 ± 0.18	3.32 ± 0.53
	Run2	50 ns	264.53 ± 0.11	268.19 ± 0.21	
	Run3	50 ns	265.43 ± 0.13	269.68 ± 0.17	
		**Average**	**265.03 ± 0.26**	**268.35 ± 0.46**	

Table S3: Data similar to Table S2 are shown for single mutations in the TBP variant P2.

**System P2/TAR**	**Runs**	**Simulation Length**	**ΔG^{free}**	**ΔG^{comp}**	**$\Delta\Delta G = (\Delta G^{comp} - \Delta G^{free})$**
R47→A47	Run1	50 ns	265.38 ± 0.16	274.91 ± 0.32	9.01 ± 0.92
	Run2	50 ns	263.77 ± 0.11	272.18 ± 0.22	
	Run3	50 ns	264.76 ± 0.13	273.85 ± 0.24	
		**Average**	**264.64 ± 0.47**	**273.65 ± 0.79**	
R49→A49	Run1	50 ns	262.66 ± 0.20	268.11 ± 0.25	5.40 ± 0.45
	Run2	50 ns	262.82 ± 0.13	268.69 ± 0.19	
	Run3	50 ns	263.92 ± 0.11	268.80 ± 0.20	
		**Average**	**263.13 ± 0.40**	**268.53 ± 0.21**	
R52→A52	Run1	50 ns	265.07 ± 0.18	269.95 ± 0.24	4.79 ± 0.52
	Run2	50 ns	265.54 ± 0.13	269.68 ± 0.25	
	Run3	50 ns	263.93 ± 0.17	269.29 ± 0.25	
		**Average**	**264.85 ± 0.48**	**269.64 ± 0.19**	

Table S4: Data similar to Table S2 are shown for single mutations in the TBP variant P3.

System P3/TAR	Runs	Simulation Length	ΔG^{free}	$(\Delta G^{comp}$	$\Delta\Delta G = (\Delta G^{comp} - \Delta G^{free})$
R47→A47	Run1	50 ns	265.38 ± 0.16	273.84 ± 0.26	9.07 ± 0.45
	Run2	50 ns	265.51 ± 0.12	274.21 ± 0.28	
	Run3	50 ns	264.17 ± 0.10	274.22 ± 0.25	
		**Average**	**265.02 ± 0.43**	**274.09 ± 0.12**	
R49→A49	Run1	50 ns	264.58 ± 0.11	270.43 ± 0.23	6.20 ± 0.37
	Run2	50 ns	264.60 ± 0.15	271.43 ± 0.21	
	Run3	50 ns	264.22 ± 0.10	270.32 ± 0.20	
		**Average**	**264.47 ± 0.12**	**270.73 ± 0.35**	
R52→A52	Run1	50 ns	264.43 ± 0.13	268.99 ± 0.30	4.60 ± 0.61
	Run2	50 ns	265.50 ± 0.14	269.65 ± 0.24	
	Run3	50 ns	265.66 ± 0.16	270.76 ± 0.20	
		**Average**	**265.20 ± 0.32**	**269.80 ± 0.52**	

Table S5: Data similar to Table S2 are shown for single mutations in the TBP variant P4.

System P4/TAR	Runs	Simulation Length	ΔG^{free}	ΔG^{comp}	$\Delta\Delta G = (\Delta G^{comp} - \Delta G^{free})$
R47→A47	Run1	50 ns	264.21 ± 0.11	273.78 ± 0.24	9.02 ± 0.27
	Run2	50 ns	264.96 ± 0.13	273.75 ± 0.30	
	Run3	50 ns	264.56 ± 0.19	273.28 ± 0.32	
		**Average**	**264.58 ± 0.22**	**273.60 ± 0.16**	
R49→A49	Run1	50 ns	264.44 ± 0.11	270.91 ± 0.19	5.97 ± 0.60
	Run2	50 ns	264.28 ± 0.11	270.76 ± 0.21	
	Run3	50 ns	264.10 ± 0.10	269.05 ± 0.24	
		**Average**	**264.27 ± 0.10**	**270.24 ± 0.59**	
R52→A52	Run1	50 ns	264.53 ± 0.22	269.50 ± 0.22	5.01 ± 0.31
	Run2	50 ns	264.98 ± 0.15	269.27 ± 0.25	
	Run3	50 ns	264.33 ± 0.14	270.08 ± 0.23	
		**Average**	**264.61 ± 0.19**	**269.62 ± 0.24**	

Table S6. Free energy data for single mutations in the P3/TAR complex. The computed relative binding free energy (ΔΔG) is reported in the second column while the experimental dissociation constants (K_d) and estimated ΔΔG values are shown in the third and fourth columns, respectively. The K_d values were converted to ΔΔG using the following expression: $\Delta\Delta G = RT \ln (K_{d2}/ K_{d1})$, where T = 293.15 K (as used in experiments), K_{d2} is the dissociation constant for a given mutant form of P3, and K_{d1} is the dissociation constant for WT P3.

**System P3/TAR**	**ΔΔG (Computed) kcal/mol**	**K_d (nM)**	**ΔΔG (Experimental) kcal/mol**
WT	-	2.5 ± 0.1	-
R47→A47	9.07 ± 0.45	1516 ± 163	3.8
R49→A49	6.20 ± 0.37	583 ± 21	3.2
R52→A52	4.60 ± 0.61	290 ± 57	2.8

Table S7: Free energy data for double/triple mutations for the TBP variant P1. The computed free energy change upon mutations in combination of two (mutants: R47A-R49A, R49A-R50A, and R47A-R50A) and three (R47A-R49A-R50A) Arg residues of P1 in solution (ΔG^{free}) and in complex (ΔG^{comp}) with TAR.

**System P1/TAR**	**Runs**	**Simulation Length**	**ΔG^{free}**	**ΔG^{comp}**	**$\Delta\Delta G = (\Delta G^{comp} - \Delta G^{free})$**
R47R49→ A47A49	Run1	50 ns	530.54 ± 0.20	546.65 ± 0.47	16.00 ± 1.08
	Run2	50 ns	529.50 ± 0.15	547.64 ± 0.29	
	Run3	50 ns	532.97 ± 0.17	546.70 ± 0.25	
		**Average**	**531.00 ± 1.03**	**547.00 ± 0.32**	
R49R52→ A49A50	Run1	50 ns	529.42 ± 0.27	541.91 ± 0.23	12.84 ± 0.58
	Run2	50 ns	528.55 ± 0.15	541.72 ± 0.22	
	Run3	50 ns	527.87 ± 0.14	540.73 ± 0.24	
		**Average**	**528.61 ± 0.45**	**541.45 ± 0.37**	
R47R50→ A47A50	Run1	50 ns	530.58 ± 0.15	542.57 ± 0.34	15.13 ± 1.54
	Run2	50 ns	529.54 ± 0.13	547.40 ± 0.25	
	Run3	50 ns	528.36 ± 0.15	543.89 ± 0.27	
		**Average**	**529.49 ± 0.64**	**544.62 ± 1.4**	
R47R49R50→ A47A49A50	Run1	50 ns	793.79 ± 0.39	815.81 ± 0.49	21.15 ± 0.67
	Run2	50 ns	793.25 ± 0.19	814.92 ± 0.35	
	Run3	50 ns	793.84 ± 0.20	813.62 ± 0.31	
		**Average**	**793.63 ± 0.19**	**814.78 ± 0.64**	

Table S8: Data similar to Table S6 are shown for double/triple mutations in P2.

**System P2/TAR**	**Runs**	**Simulation Length**	**ΔG^{free}**	**ΔG^{comp}**	**$\Delta\Delta G = (\Delta G^{comp} - \Delta G^{free})$**
R47R49→ A47A49	Run1	50 ns	530.28 ± 0.16	544.08 ± 0.31	13.01 ± 0.86
	Run2	50 ns	530.70 ± 0.15	543.55 ± 0.32	
	Run3	50 ns	532.68 ± 0.21	545.05 ± 0.33	
		**Average**	**531.22 ± 0.74**	**544.23 ± 0.44**	
R49R52→ A49A52	Run1	50 ns	529.92 ± 0.25	542.26 ± 0.42	12.10 ± 0.50
	Run2	50 ns	528.83 ± 0.19	541.40 ± 0.29	
	Run3	50 ns	530.22 ± 0.14	541.63 ± 0.30	
		**Average**	**529.66 ± 0.43**	**541.76 ± 0.25**	
R47R52→ A47A52	Run1	50 ns	530.25 ± 0.24	544.41 ± 0.44	13.43 ± 0.97
	Run2	50 ns	529.53 ± 0.18	543.08 ± 0.25	
	Run3	50 ns	528.75 ± 0.19	541.43 ± 0.28	
		**Average**	**529.54 ± 0.46**	**542.97 ± 0.86**	
R47R49R52→ A47A49A52	Run1	50 ns	798.20 ± 0.27	816.60 ± 0.61	19.38 ± 1.22
	Run2	50 ns	794.59 ± 0.20	814.46 ± 0.38	
	Run3	50 ns	796.37 ± 0.21	816.20 ± 0.33	
		**Average**	**796.37 ± 1.04**	**815.75 ± 0.65**	

Table S9: Data similar to Table S6 are shown for double/triple mutations in P3.

**System P3/TAR**	**Runs**	**Simulation Length**	**ΔG^{free}**	**ΔG^{comp}**	**ΔΔG = (ΔG^{comp} -ΔG^{free})**
R47R49→ A47A49	Run1	50 ns	530.64 ± 0.18	545.34 ± 0.45	13.53 ± 1.00
	Run2	50 ns	530.85 ± 0.37	545.73 ± 0.28	
	Run3	50 ns	531.87 ± 0.17	542.87 ± 0.28	
		**Average**	**531.12 ± 0.38**	**544.65 ± 0.89**	
R49R52→ A49A52	Run1	50 ns	529.87 ± 0.22	542.09 ± 0.41	11.01 ± 1.06
	Run2	50 ns	531.17 ± 0.18	542.56 ± 0.23	
	Run3	50 ns	530.06 ± 0.18	539.49 ± 0.23	
		**Average**	**530.37 ± 0.33**	**541.38 ± 0.95**	
R47R52→ A47A52	Run1	50 ns	528.58 ± 0.16	540.60 ± 0.55	11.49 ± 1.59
	Run2	50 ns	529.47 ± 0.31	543.57 ± 0.30	
	Run3	50 ns	529.91 ± 0.17	538.25 ± 0.28	
		**Average**	**529.32 ± 0.39**	**540.81 ± 1.54**	
R47R49R52→ A47A49A52	Run1	50 ns	797.54 ± 0.37	819.49 ± 0.90	20.17 ± 2.61
	Run2	50 ns	794.17 ± 0.31	817.36 ± 0.77	
	Run3	50 ns	795.80 ± 0.37	811.17 ± 0.41	
		**Average**	**795.84 ± 0.79**	**816.01 ± 2.49**	

Table S10: Data similar to Table S6 are shown for double/triple mutations in P4.

**System P4/TAR**	**Runs**	**Simulation Length**	**ΔG^{free}**	**ΔG^{comp}**	**ΔΔG = (ΔG^{comp} - ΔG^{free})**
R47R49→ A47A49	Run1	50 ns	530.23 ± 0.18	546.65 ± 0.45	14.09 ± 0.72
	Run2	50 ns	531.04 ± 0.14	542.76 ± 0.21	
	Run3	50 ns	529.19 ± 0.17	543.32 ± 0.22	
		**Average**	**530.15 ± 0.53**	**544.24 ± 1.2**	
R49R52→ A49A52	Run1	50 ns	528.30 ± 0.19	538.74 ± 0.41	12.01 ± 0.80
	Run2	50 ns	526.90 ± 0.16	540.35 ± 0.25	
	Run3	50 ns	527.01 ± 0.17	539.74 ± 0.27	
		**Average**	**527.60 ± 0.70**	**539.61 ± 0.47**	
R47R52→ A47A52	Run1	50 ns	530.24 ± 0.24	543.13 ± 0.36	14.42 ± 0.74
	Run2	50 ns	530.15 ± 0.18	543.88 ± 0.29	
	Run3	50 ns	528.52 ± 0.19	545.17 ± 0.28	
		**Average**	**529.64 ± 0.56**	**544.06 ± 0.49**	
R47R49R52→ A47A49A52	Run1	50 ns	794.87 ± 0.29	813.56 ± 0.55	19.84 ± 1.04
	Run2	50 ns	793.35 ± 0.22	812.03 ± 0.41	
	Run3	50 ns	791.50 ± 0.19	813.64 ± 0.33	
		**Average**	**793.24 ± 0.97**	**813.08 ± 0.52**	

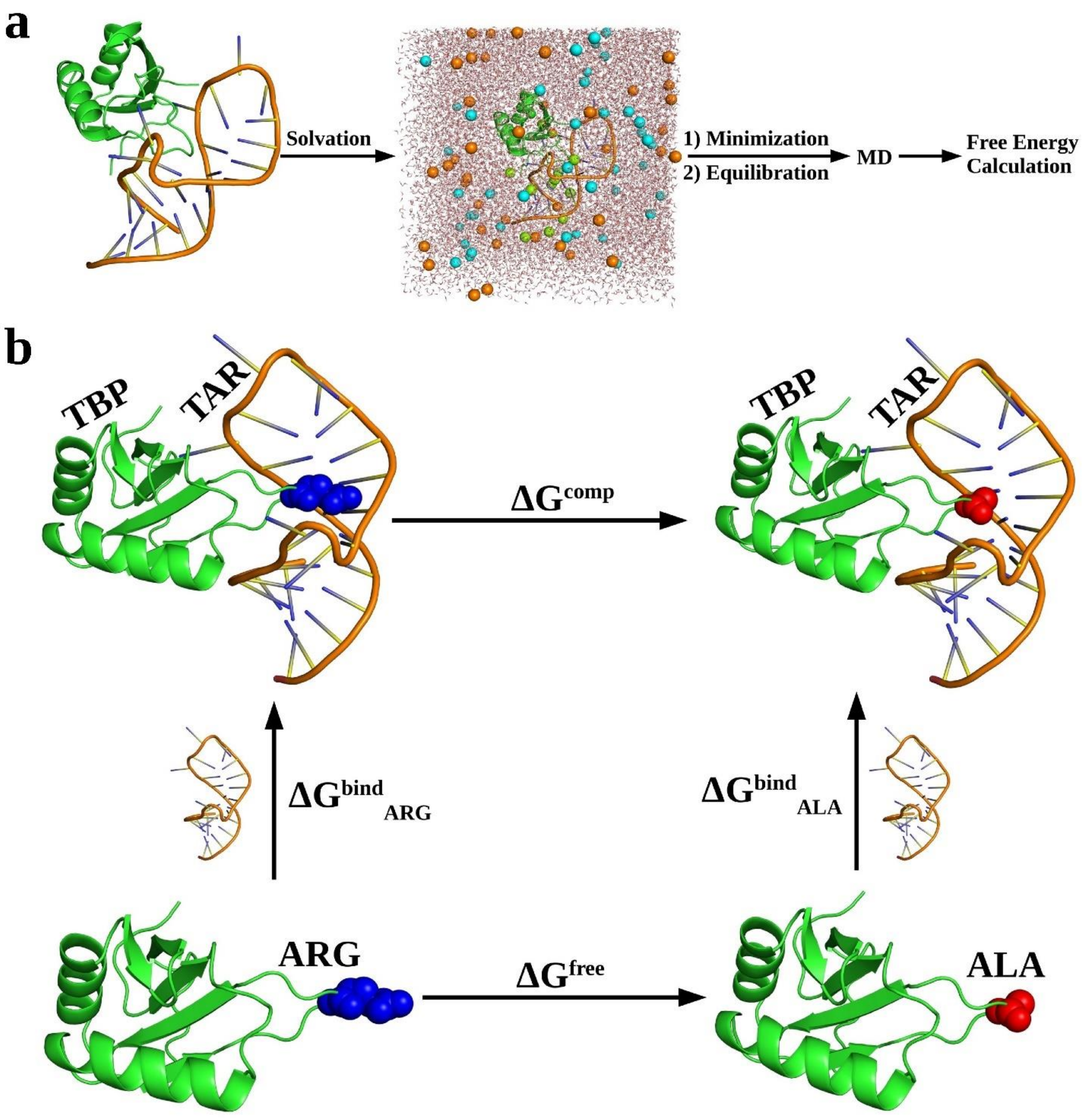

Fig. S1: (a) The steps taken for system setup and MD simulations are highlighted. (b) The thermodynamic cycle describing the relative binding free energy (ΔΔG) of a mutation in a given TBP. The horizontal arms of the thermodynamic cycle correspond to the alchemical transformation of the wild-type TBP into a mutant TBP in the complex (upper horizontal arm) and in solution (bottom horizontal arm). The free energy calculations were used to compute the free energy changes (ΔG^{comp}, ΔG^{free}).

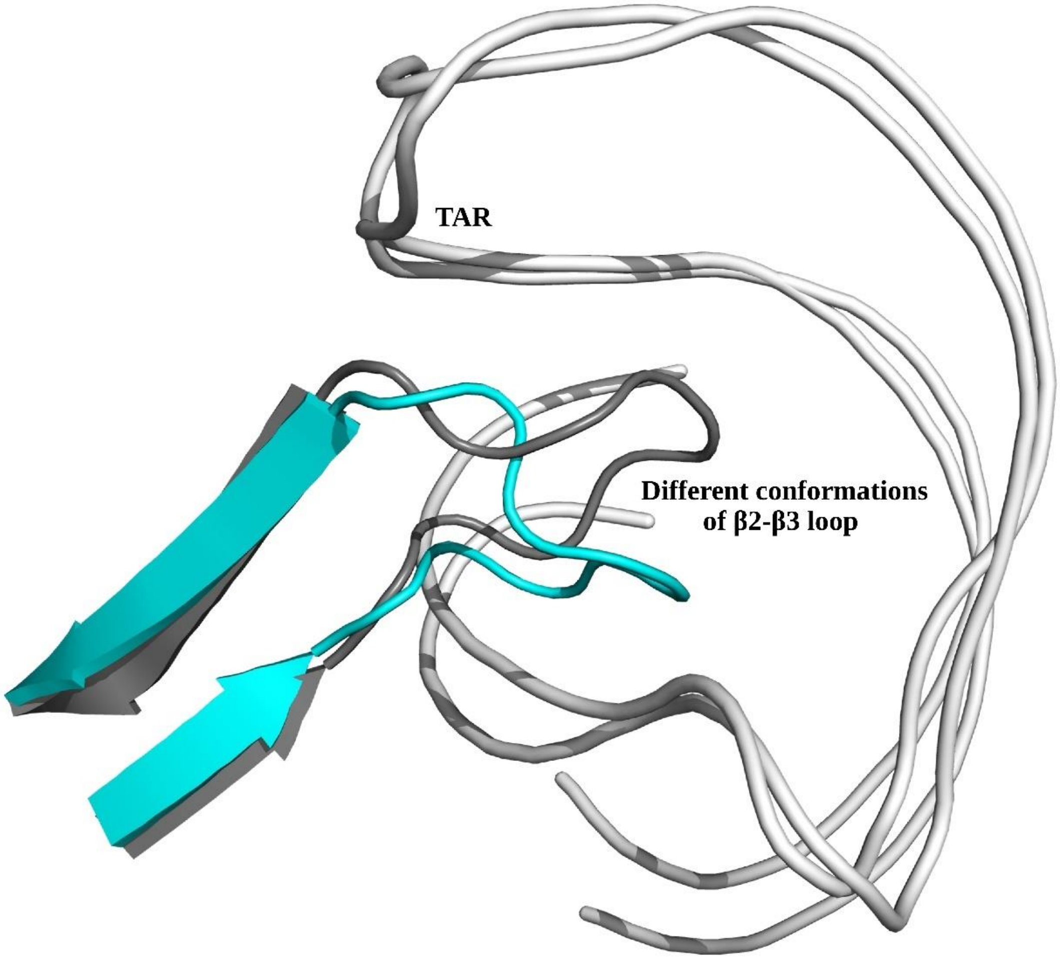

Fig. S2: For the P3/TAR complex, a comparison of the β2-β3 loop conformation from the crystal structure (gray color) and from MD (cyan color) upon reverse transformation (A→R) of A47R and A49R.

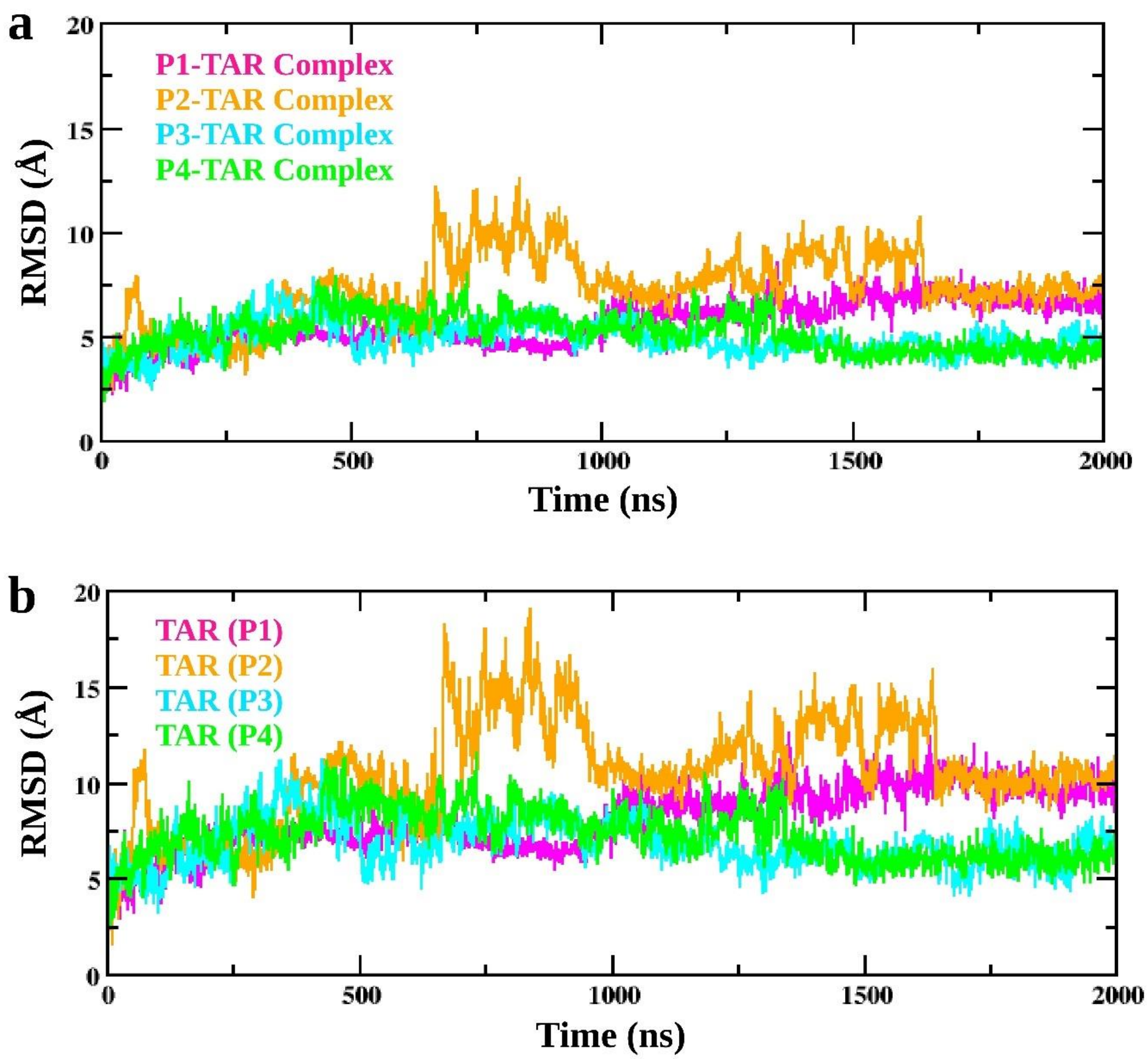

Fig. S3: The root mean squared deviation (RMSD) of all heavy atoms in each TBP-TAR complex (panel a) and the RMSD for only TAR RNA in each complex (panel b).

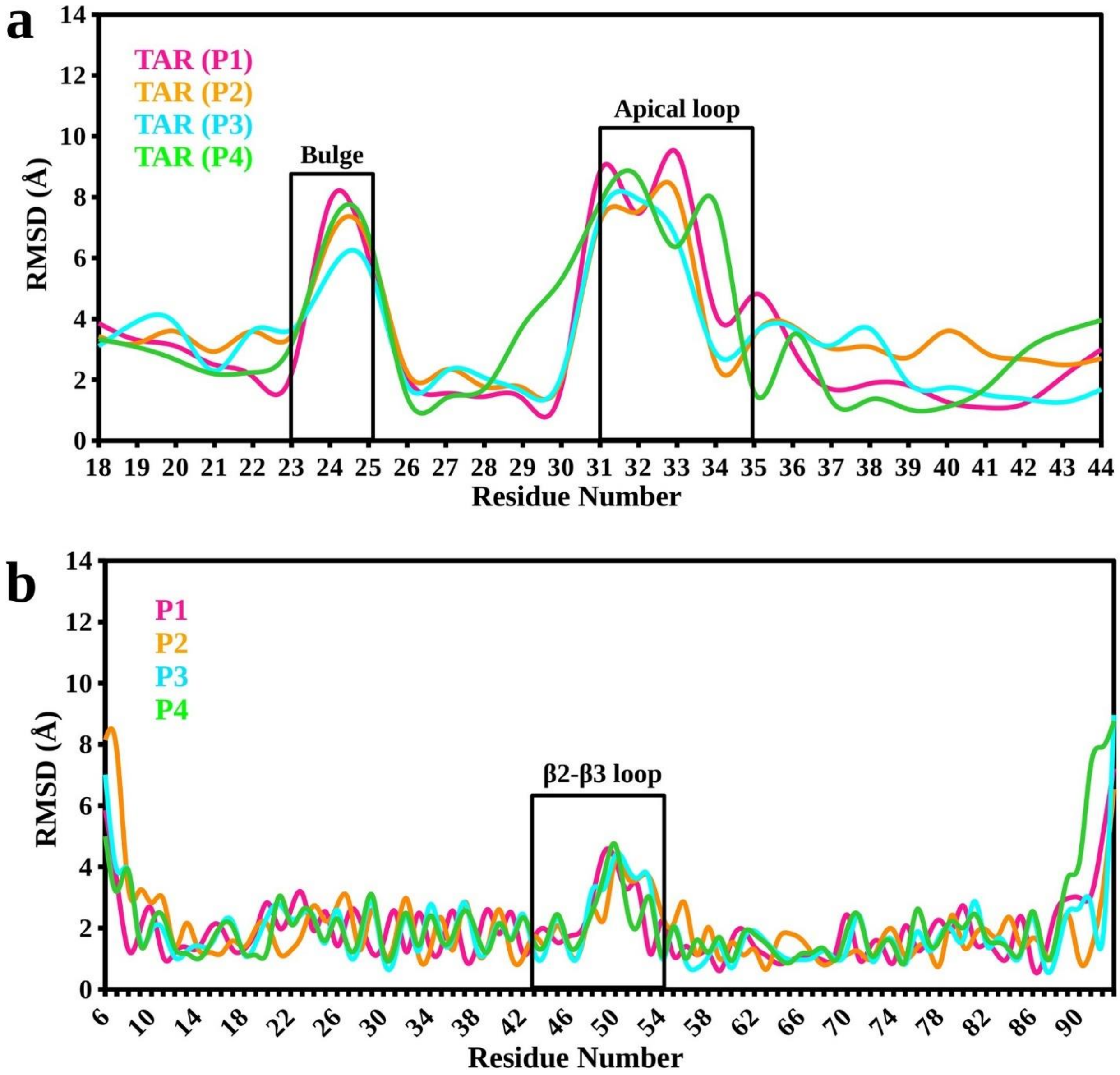

Fig. S4: The average RMSD of each residue of TAR (panel a) and TBPs (panel b) from different TBP-TAR complexes.

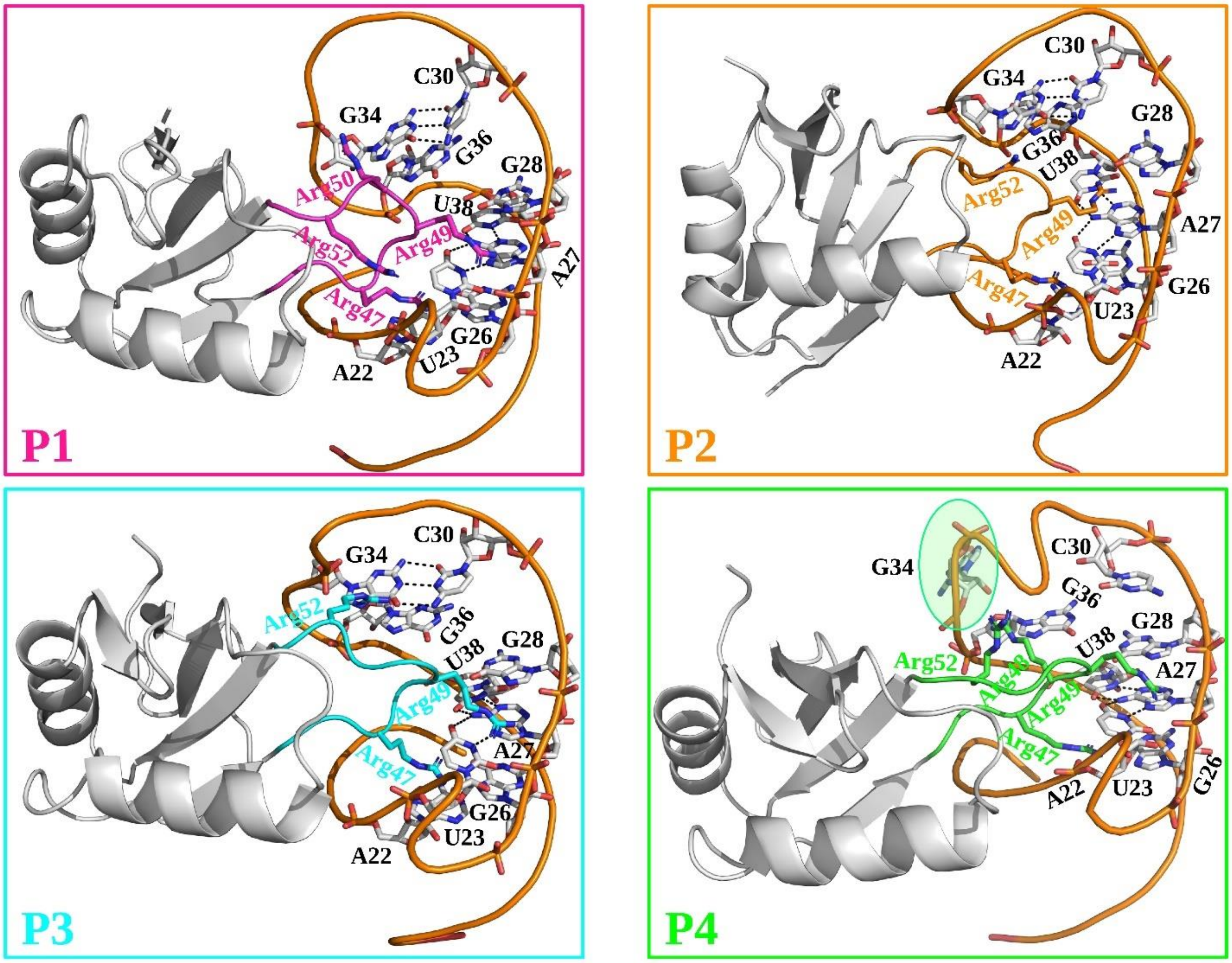

Fig. S5: MD averaged structures of the TBP-TAR complexes. Different complexes are colored and labeled in unique colors. The key amino acids and nucleotides are represented with sticks and their interactions are highlighted by black dotted lines. The nucleotide GUA34 (highlighted in a green oval) is oriented away from the major groove of TAR and disrupts the canonical CYT30-GUA34 base pairing in the P4/TAR complex.

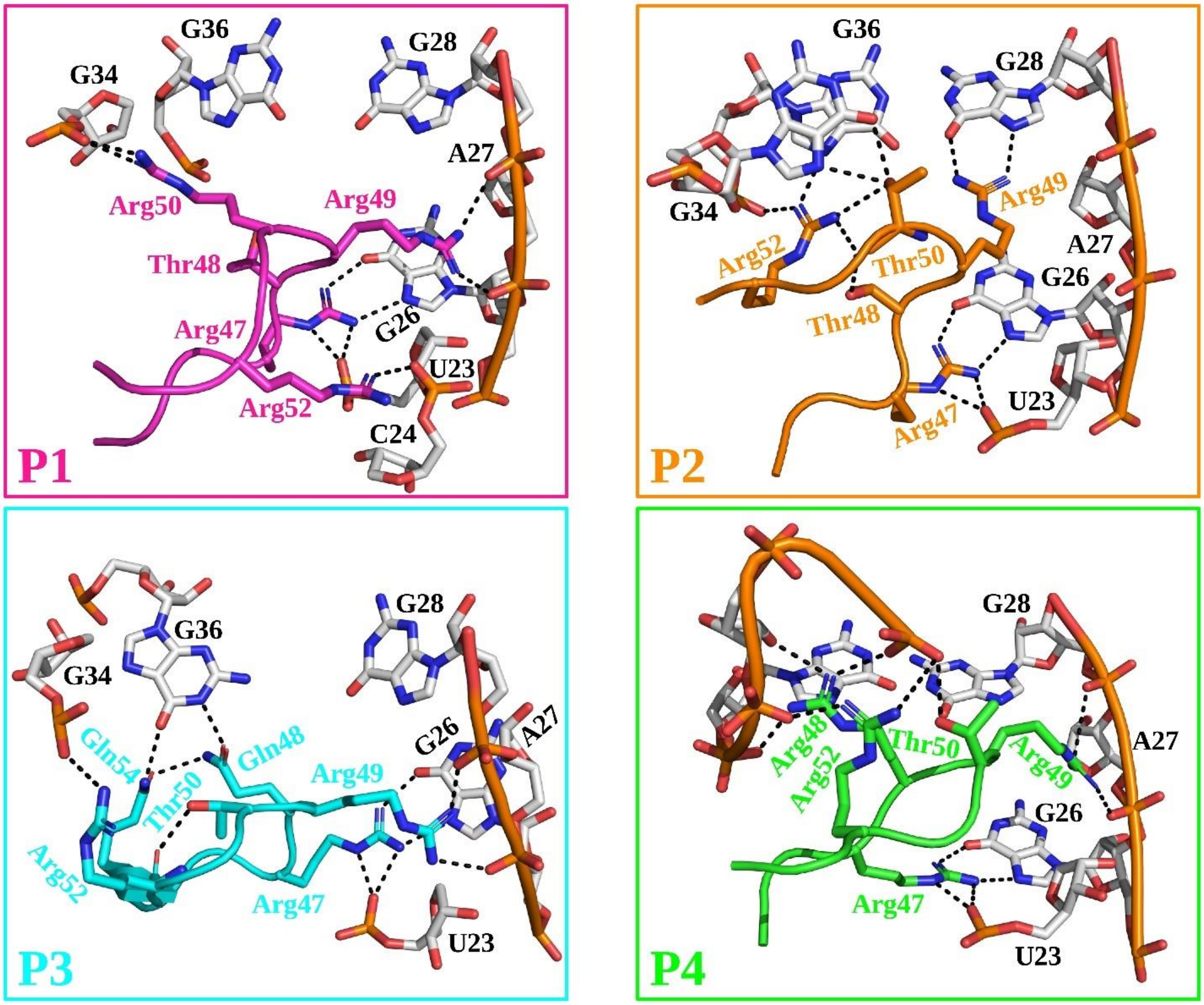

Fig. S6: A zoomed view of the TBP-TAR binding pocket from MD simulations of different TBP-TAR complexes: (a) P1/TAR, (b) P2/TAR, (c) P3/TAR, and (d) P4/TAR complex. The key amino acids and TAR nucleotides are represented by sticks. The nucleobase part of some nucleotides has not been shown for clarity. The interactions in the major groove of TAR are shown with black dotted lines.

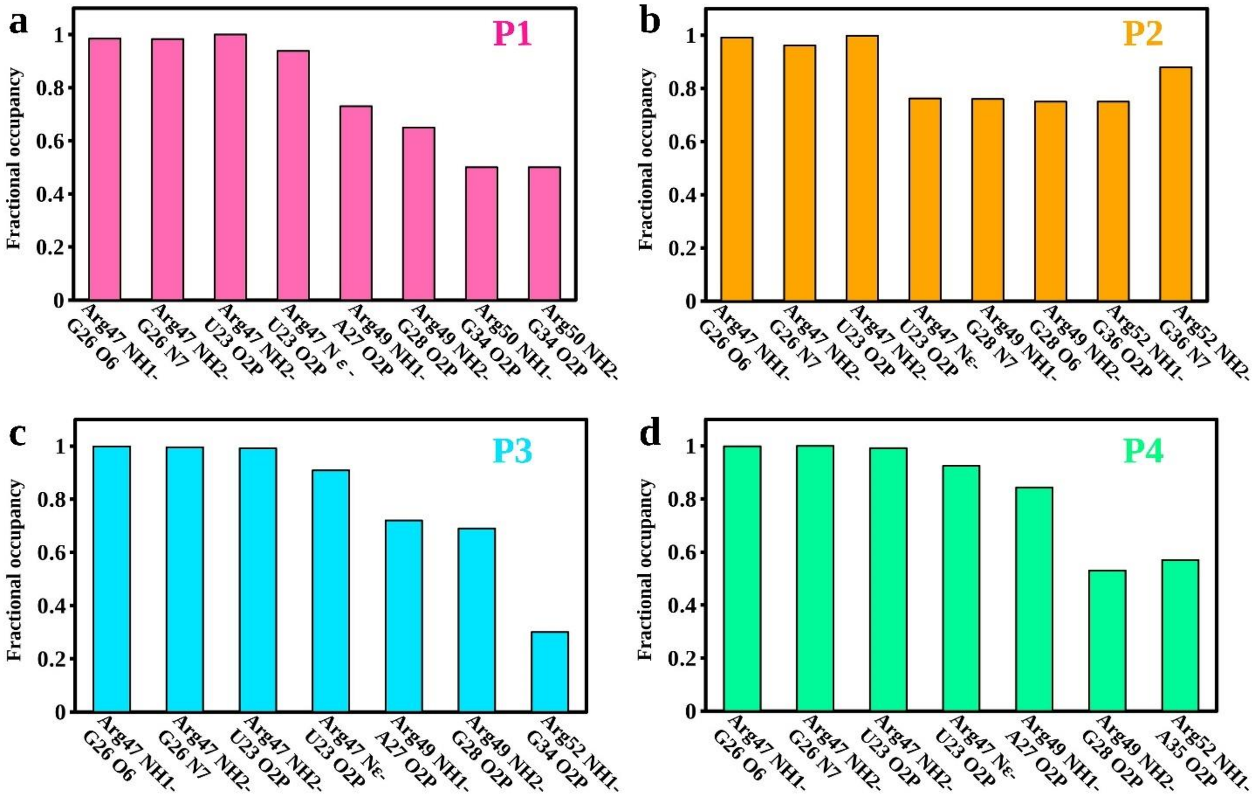

Fig. S7: The fractional occupancies of interactions of key Arg residues observed in the major groove of the TBP-TAR complexes. These data are derived from MD trajectories of (a) P1/TAR, (b) P2/TAR, (c) P3/TAR, and (d) P4/TAR complex. The fractional occupancy is the fraction of simulation frames, sampled at 1 ns, in which a specific interaction is occupied. We determined the hydrogen bond occupancy using a cutoff of 3.5 Å for heavy atoms (oxygen and nitrogen).

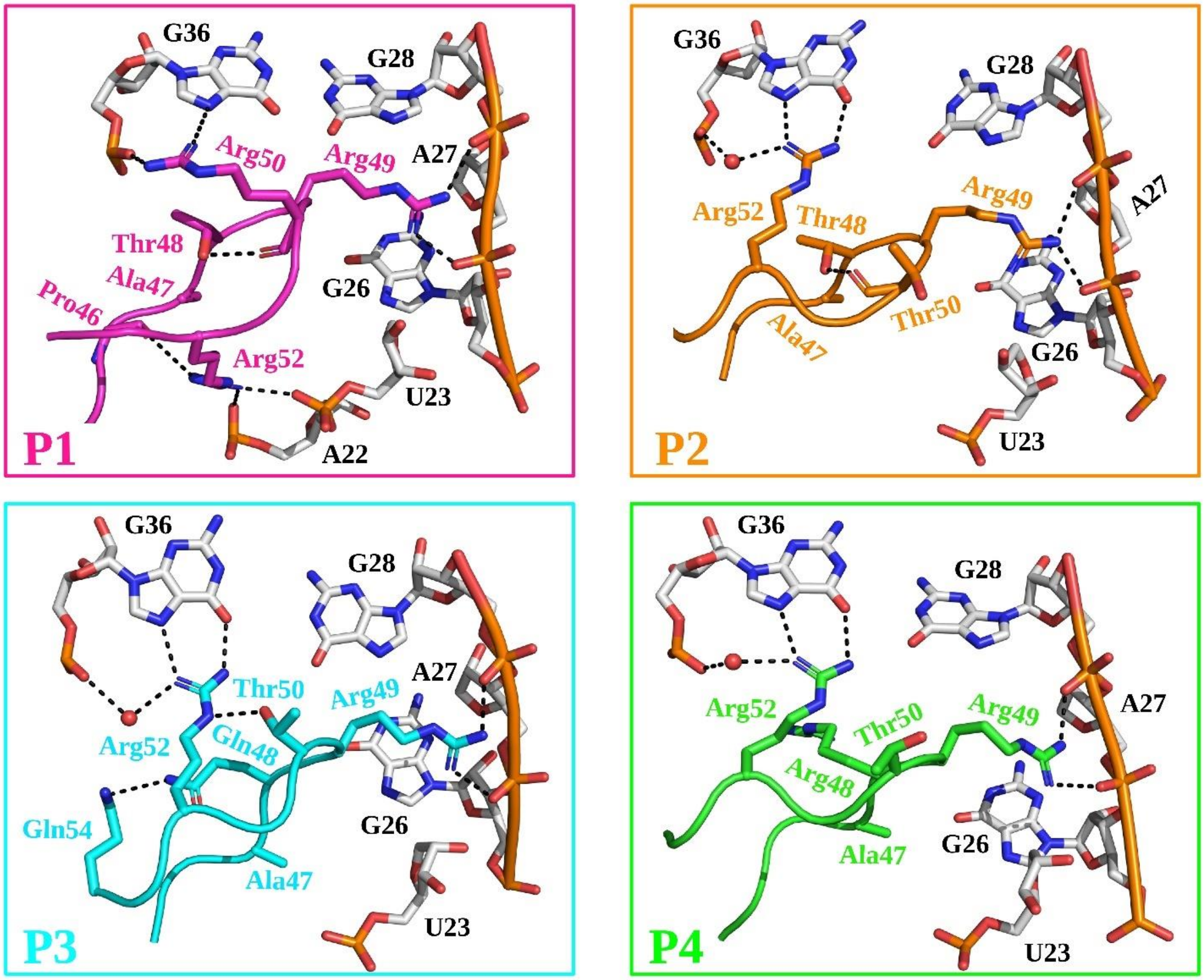

Fig. S8. Structural insights from MD simulations of the R47A mutated TBP-TAR complexes: (a) P1/TAR, (b) P2/TAR, (c) P3/TAR, and (d) P4/TAR. The β2-β3 loop is shown as a cartoon and the amino acid residues interacting with the TAR nucleotides are shown as sticks. The hydrogen atoms and the nucleobases of ADE22, URI23 and A2DE7 are not shown for clarity. The key interactions are highlighted by black dotted lines. Water molecules are indicated by a red sphere.

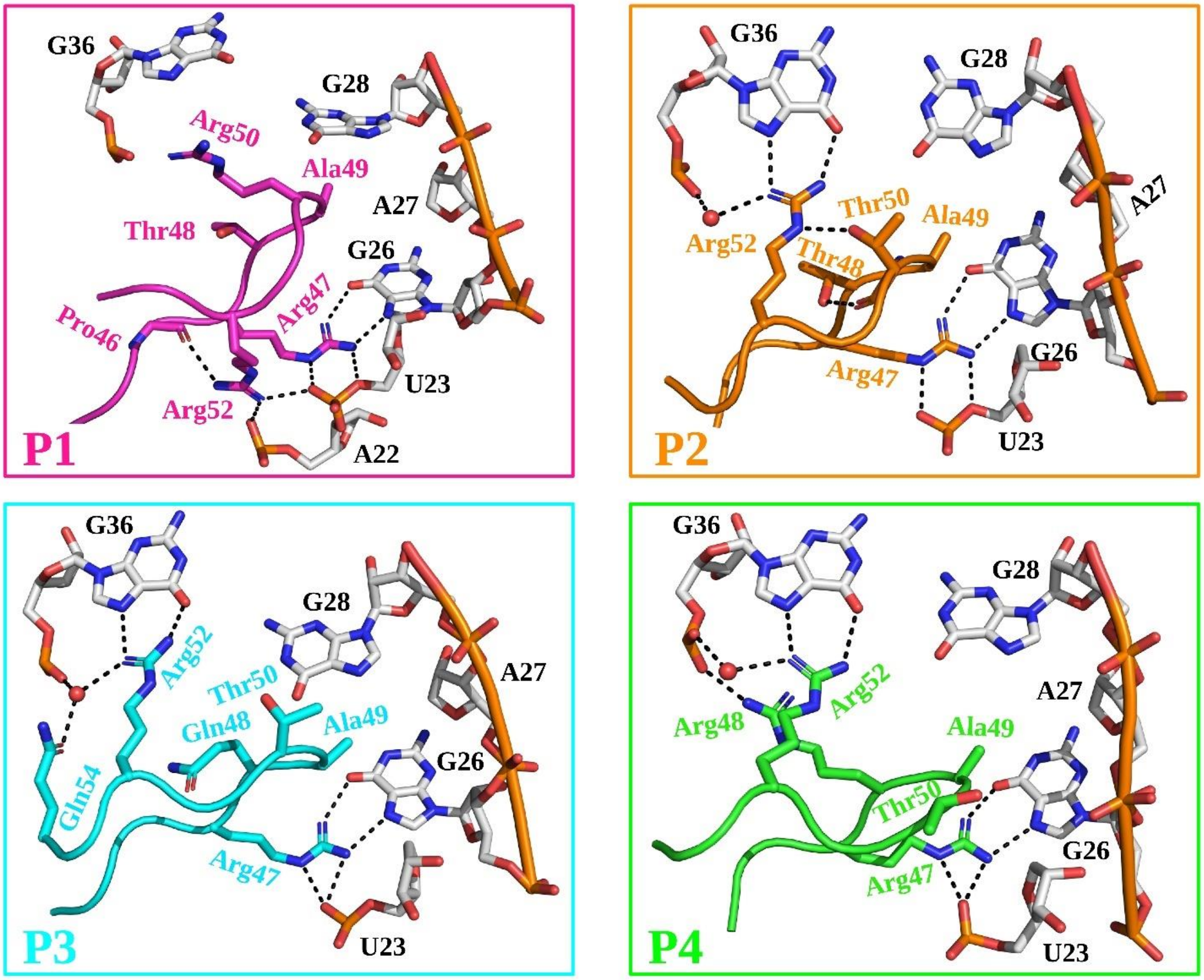

Fig. S9. Snapshots similar to Fig. S8 are shown for the R49A mutation in TBP-TAR complexes.

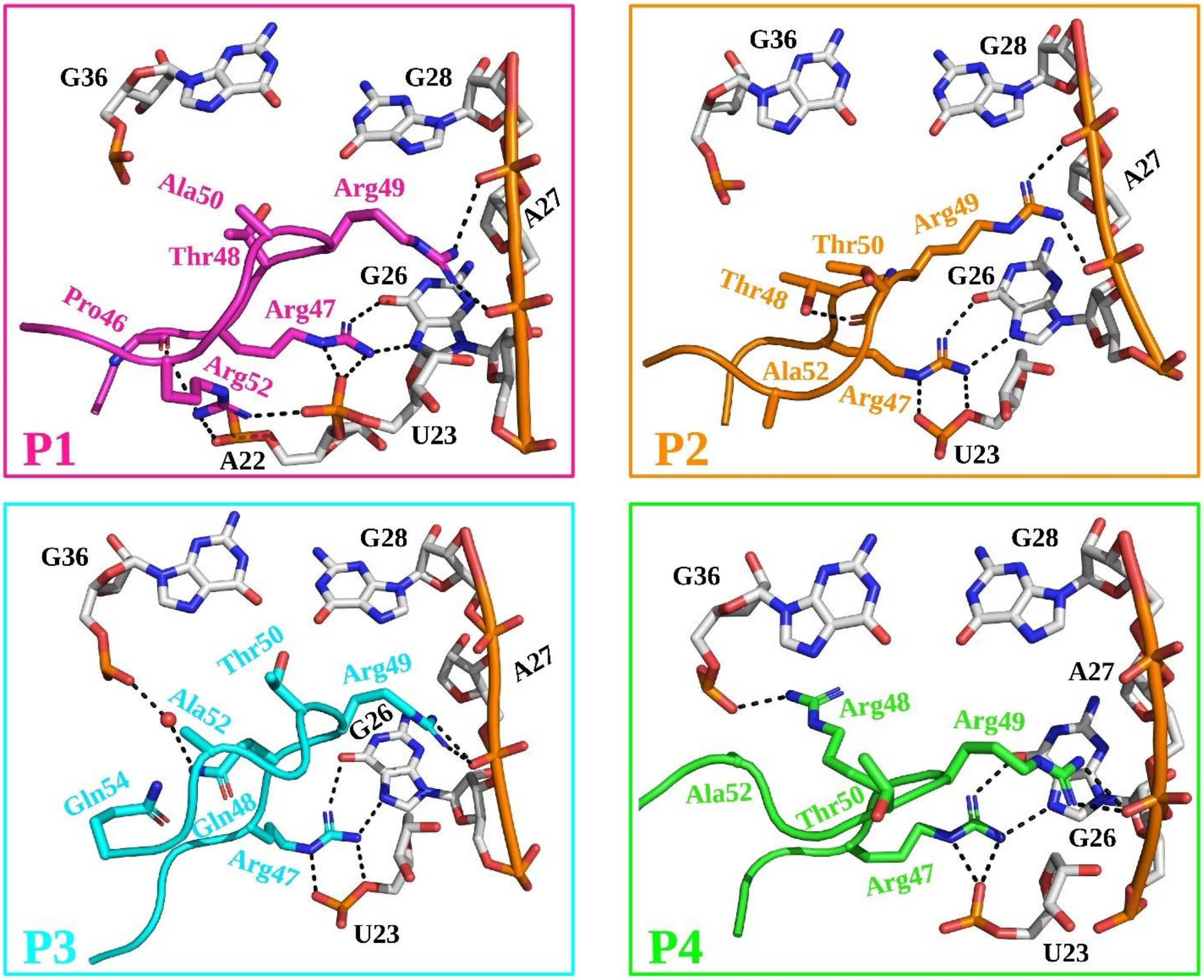

Fig. S10. Snapshots similar to Fig. S8 are shown for the R52A (R50A in case of P1) mutation in TBP-TAR complexes.

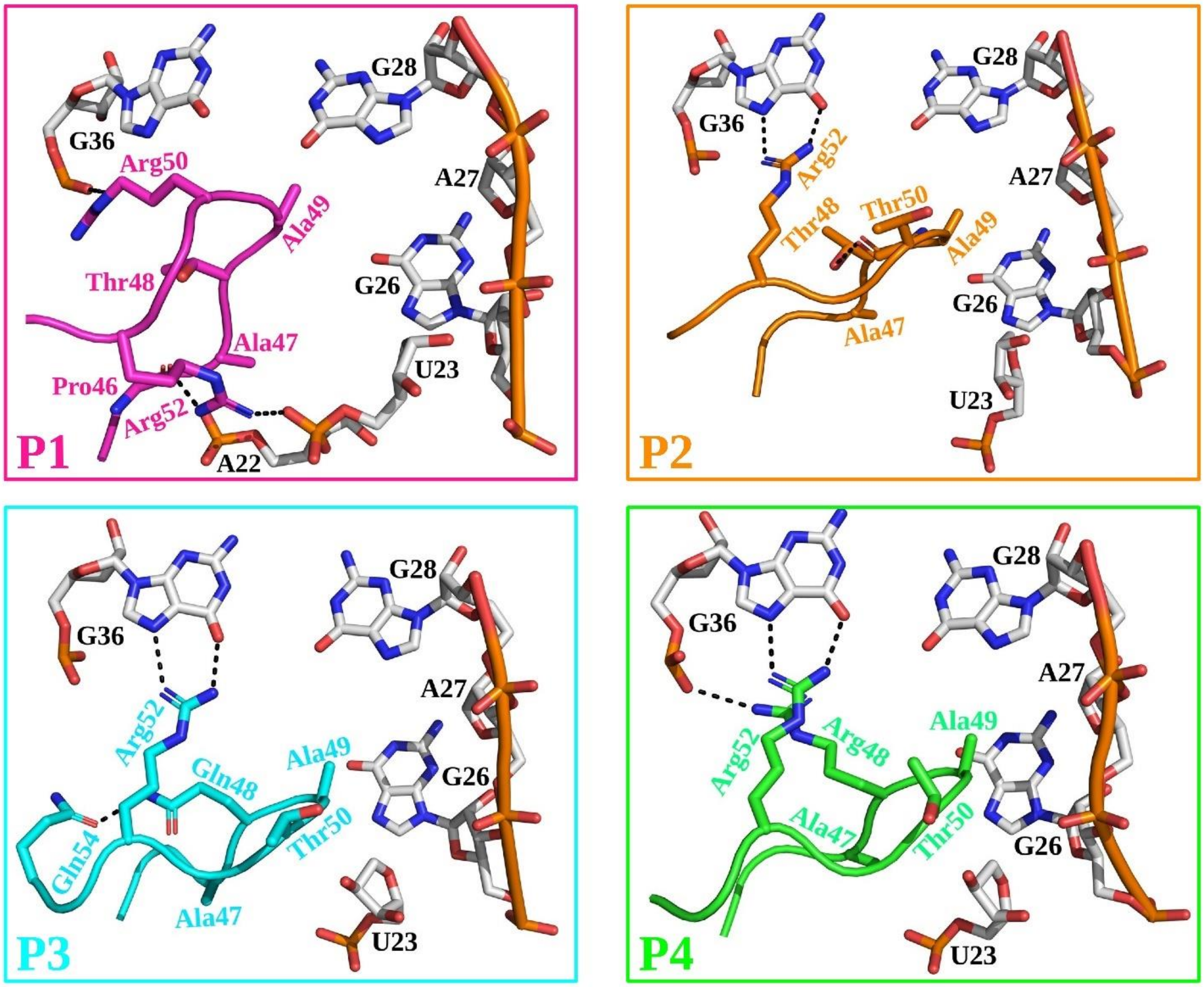

Fig. S11. Snapshots similar to Fig. S8 are shown for the R47A-R49A mutations in TBP-TAR complexes.

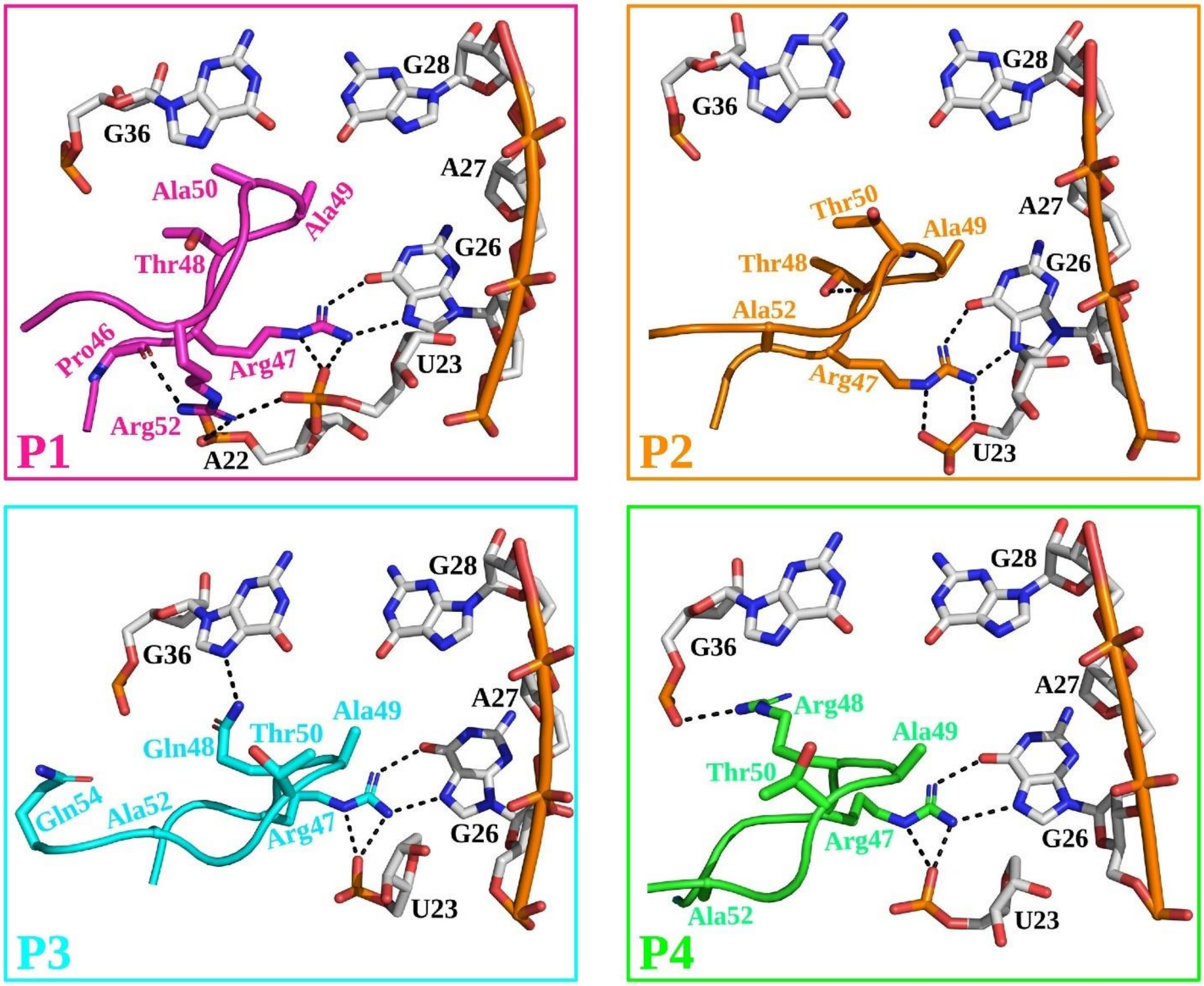

Fig. S12. Snapshots similar to Fig. S8 are shown for the R49A-R52A (R50A in case of P1) mutations in TBP-TAR complexes.

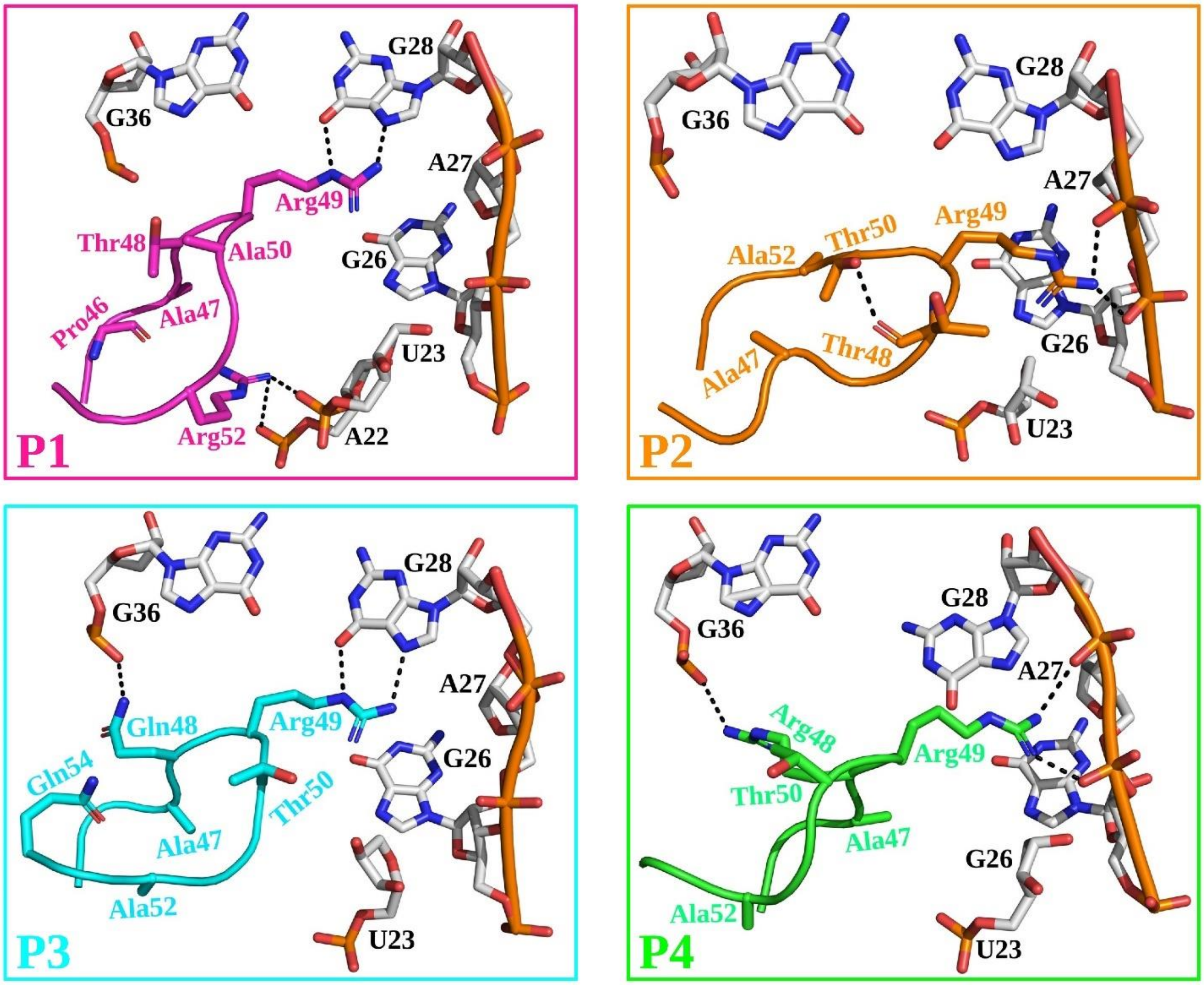

Fig. S13. Snapshots similar to Fig. S8 are shown for the R47A-R52A (R50A in case of P1) mutations in TBP-TAR complexes.

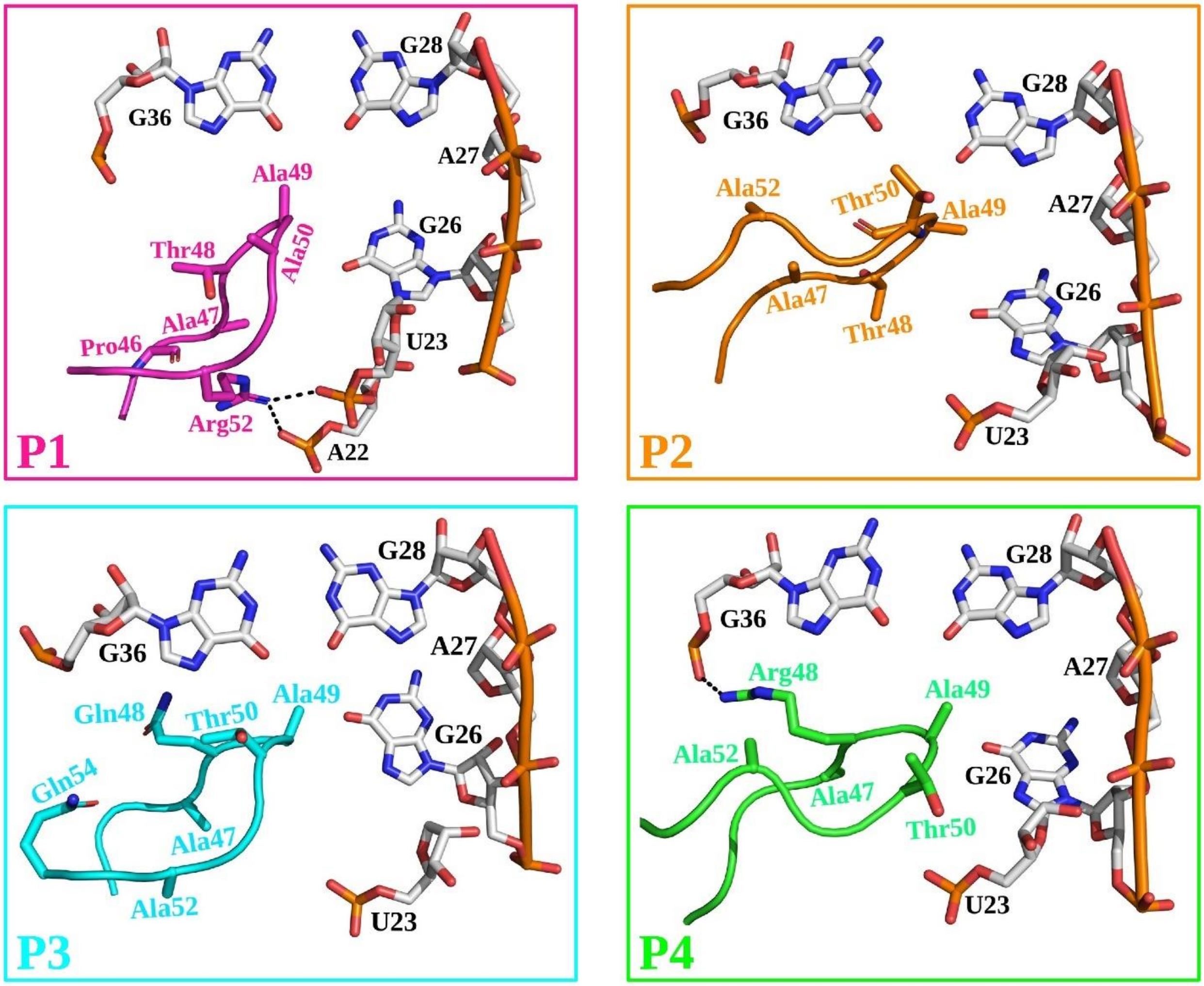

Fig. S14. Snapshots similar to Fig. S8 are shown for the R47A-R49A-R52A (R50A in case of P1) mutations in TBP-TAR complexes.
